# Mutational signatures representative transcriptomic perturbations in hepatocellular carcinoma

**DOI:** 10.3389/fgene.2022.970907

**Published:** 2022-08-23

**Authors:** Qiong Wu, Lingyi Wang, Stephen Kwok-Wing Tsui

**Affiliations:** ^1^ School of Biomedical Sciences, The Chinese University of Hong Kong, Shatin, New Territories, Hong Kong SAR, China; ^2^ Department of Paediatrics, Prince of Wales Hospital, The Chinese University of Hong Kong, Shatin, New Territories, Hong Kong SAR, China

**Keywords:** multiomics, mutational signature, miRNA, lncRNA, ceRNA network

## Abstract

Hepatocellular carcinoma (HCC) is a primary malignancy with increasing incidence and poor prognosis. Heterogeneity originating from genomic instability is one of the critical reasons of poor outcomes. However, the studies of underlying mechanisms and pathways affected by mutations are still not intelligible. Currently, integrative molecular-level studies using multiomics approaches enable comprehensive analysis for cancers, which is pivotal for personalized therapy and mortality reduction. In this study, genomic and transcriptomic data of HCC are obtained from The Cancer Genome Atlas (TCGA) to investigate the affected coding and non-coding RNAs, as well as their regulatory network due to certain mutational signatures of HCC. Different types of RNAs have their specific enriched biological functions in mutational signature-specific HCCs, upregulated coding RNAs are predominantly associated with lipid metabolism-related pathways, and downregulated coding RNAs are enriched in axonogenesis for tumor microenvironment generation. Additionally, differentially expressed miRNAs are inclined to concentrate in cancer-related signaling pathways. Some of these RNAs also serve as prognostic factors that help predict the survival outcome of HCCs with certain mutational signatures. Furthermore, deregulation of competing endogenous RNA (ceRNA) regulatory network is identified, which suggests a potential therapy *via* interference of miRNA activity for mutational signature-specific HCC. This study proposes a projection approach to reduce therapeutic complexity from genomic mutations to transcriptomic alterations. Through this method, we identify genes and pathways critical for mutational signature-specific HCC and further discover a series of prognostic markers indicating patient survival outcome.

## Introduction

As one of the most aggressive malignancies, HCC has the second highest cancer mortality rate due to the limited therapeutic options available (Cancer Genome Atlas Research Network, 2017). Despite it is more commonly found in Asia and Africa, its incident rate has arisen in the United States and Europe with unique HCC etiologies recently (Hashem and Andrew, 1999; Hashem, 2004; Jessica et al., 2004). A series of etiologic agents have been identified for HCC, such as hepatitis virus infection and non-alcoholic fatty liver disease (NAFLD), however, the molecular pathogenesis remains unclear (Snorri and Joe, 2002; Ju and Snorri, 2004).

Mutations are ubiquitous in cancer and accumulated numerous genetic alterations could lead to a growth advantage to tumor cells (Francisco et al., 2020). From the decade studies, mutations initiate HCC in the formation of combinative alterations of specific mutagenesis processes (Miryam et al., 2020). Based on this postulation, the concept of mutational signatures as well as predictive genomic biomarkers of response to immunotherapy are introduced to HCC studies (Mark et al., 2017). In theory, the recognition of tumor cells by T cell is largely dependent on the level of mutational complexity. A higher degree of complexity could potentially lead to more beneficial effect when immunotherapy is given to an HCC patient (Chan et al., 2019). However, more in-depth studies are necessary to elucidate the effect of mutations in facilitating HCC development.

With the advent of multiomics technology development, increasing number of integrative studies has drawn more attention on the impact of mutations during cancer development (Abel et al., 2013). It is now commonly accepted that genetic aberrations directly or indirectly trigger the changes in transcriptome, protein activities, and functional pathways, which eventually promote cell proliferation and growth in cancers, including glioblastoma, ovarian, and lung squamous (Sam et al., 2012; Evan et al., 2013; Jack and Jian, 2014; Peilin and Zhao, 2017).

Furthermore, among transcriptomic products, non-coding RNAs (ncRNAs), such as microRNA (miRNA) and long non-coding RNA (lncRNA), that contain little or no observable protein coding capacity (Eleni et al., 2018), play crucial roles in regulating numerous biological functions such as post-transcriptional modification, chromatin remodeling, and signal transduction (Eleni et al., 2018).

In the past decade, increasing evidence have supported the hypothesis of competitive endogenous RNA (Margaret et al., 2007; Daniel et al., 2010; Jiayi et al., 2010; Laura et al., 2010; Zina et al., 2011), which describes the competitive relationships between some RNAs through their shared miRNAs by the common binding site at 3′ end. Target genes of the shared miRNA are able to regulate each other indirectly and alter the miRNA function through competitive communications (Yvonne et al., 2014). To date, varies of miRNAs and lncRNAs have been identified in HCC (Maryam et al., 2018; Xin et al., 2018). For example, the expression of mir-1269 has been revealed positively correlated with HCC tumor nodes, metastasis, portal vein tumor embolus and tumor capsular infiltration. In addition, the overexpression of lncRNA HULC reported in HCC corresponds to promote HCC growth, metastasis and drug resistance. However, the relationship between mutational changes and transcriptomic alterations of both coding and non-coding genes requires further investigation, such as the effects of the mutational signatures on RNA expression and the regulatory network among ceRNAs.

In this study, we aim to identify the regulatory mechanisms of HCC among multiple omics, including mutational signatures, mRNA, miRNA, lncRNA, and their ceRNA network. In addition, this study provides a projection from complicated genomic alterations to transcriptomic changes to enhance the possibility of clinical practice. Furthermore, our approach is also applicable to other diseases with heterogenous mutational landscapes in obtaining the pathogenic targets and mechanisms.

## Materials and methods

### DNA mutational data preparation and signature detection

Mutation information derived from whole exon sequencing (WES) and corresponding clinical data of HCC samples from 374 hepatocellular tumors and 50 tumor adjacent non-tumor samples were obtained from TCGA database (Cancer Genome Atlas Research Network, 2017) (Supplementary Materials). Detection of HCC mutational signatures were performed among 374 HCC tumor samples. R package maftools (v3.14) (Anand et al., 2018) was used to explore and visualize the somatic variant profile in HCC, including the HCC specific COSMIC mutational signatures of single base substitutions (SBSs). For the concrete procedure, “estimateSignatures” was utilized to identify the variant signature of HCC, and “somaticInteractions” was conducted to detect the co-occurred mutations in HCC samples. Finally, “mafSurvival” of maftools (v3.14) was performed to detect the survival outcome of significant mutations in HCC.

### Transcriptomic data processing and analysis

HCC RNA-seq data for protein-coding genes, miRNAs and lncRNAs also were downloaded from TCGA with corresponding clinical information of the same samples (Cancer Genome Atlas Research Network, 2017). For expression profiles, the pipeline limma-voom of R package limma (v3.14) (Matthew et al., 2015) was used to identify the differentially expressed genes (DEGs), including protein-coding genes, miRNA and lncRNAs. Then, we specified false discovery rate (FDR) adjusted *p*-value < 0.05 and |log2 (fold change) | > 1 as the threshold to identify significant DEGs for downstream analysis. R packages of ggthemes (v4.2.4) (https://github.com/jrnold/ggthemes) and ggpubr (v0.4.0) (https://rpkgs.datanovia.com/ggpubr/) were recruited for the visualization of DEGs in volcano plot.

Database mirDIP (v5.0.2.2) was used to detect the information of miRNAs and their target genes of both mRNAs and lncRNAs (Tomas et al., 2018). We used the strictest Score Class “Very High” and confirmed evidence from at least ten of source databases (bitargeting_May_2021, Cupid, MBStar, MirAncesTar, miranda_May_2021, miRbase, mirCoX, miRDB_v6, mirmap_May_2021, MiRNATIP, MirTar2, miRTar2GO, mirzag, MultiMiTar, PACCMIT, PITA_May_2021, RNA22, rnahybrid_May_2021, and TargetScan_v7_2) as the criteria to filter miRNA-target gene pairs. Finally, the paired relationships were visualized in Venn plot using R package eulerr (v 6.1.1) (https://github.com/jolars/eulerr, https://jolars.github.io/eulerr/) and network-based format by miRNet (v2.0) (Le et al., 2020).

### Biological function analysis

Those co-mutated genes with *p*-values less than 0.1 were chosen as the candidate genes for downstream protein-protein interaction (PPI) network construction. Based on PPI network information provided by STRING database, we analyzed the relationships among these genes using R package STRINGdb (v3.13). DEGs of protein-coding genes and target genes of differentially expressed miRNAs was used R package “org.Hs.eg.db” (v 3.14.0) (Carlson et al., 2019) to convert gene IDs to Entrez IDs, followed by R packages of gprofiler2 (v 0.2.1) (Liis et al., 2020), enrichplot (v1.14.1) (Yu, 2021), ReactomePA (v1.38.0) (Yu and He, 2016), clusterProfiler (v4.2.0) (Yu et al., 2012) and website of Enrichr to conduct functional enrichment analysis *via* databases of GO, KEGG and GSEA. Finally, we used R package ggplot2 (v 3.3.5) (Hadley, 2016) to visualize the analysis results.

### Survival analysis

For the potential biomarkers of HCC with significant mutational signatures, such as protein-coding genes and miRNA target genes, we investigated whether they could act as prognosis indicators. We used the Kaplan-Meier curve and log-rank tests to evaluate the difference in overall survival time by R package survival (v 3.2–13) (https://github.com/therneau/survival) and survminer (v 0.4.9) (https://rpkgs.datanovia.com/survminer/index.html).

## Results

### Mutational signatures identification in HCC

**FIGURE 1:**
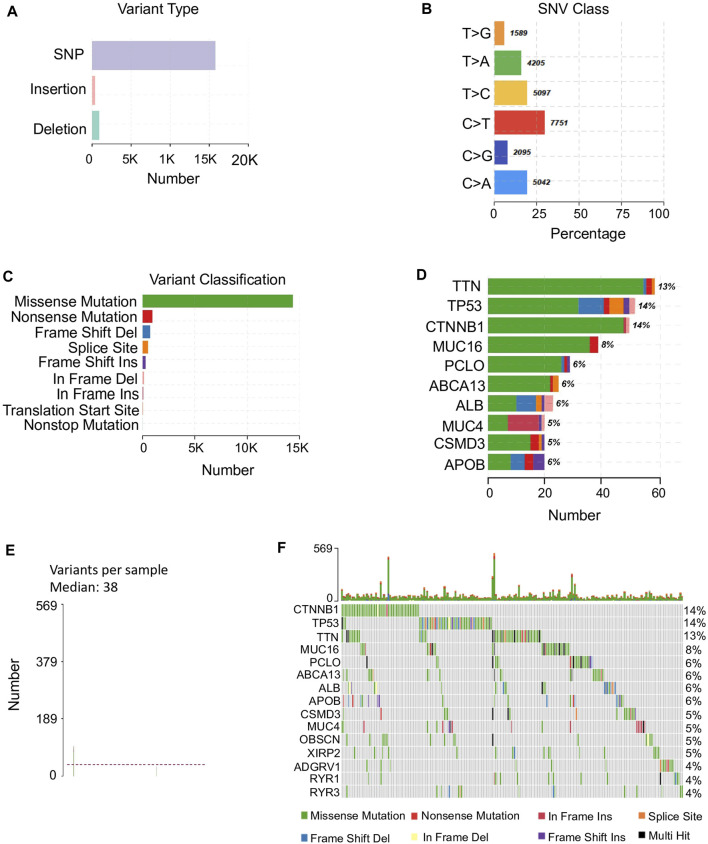
Mutation profiles in HCC. **(A)** Major variant types found in HCC representing in y-axis and the corresponding count representing in x-axis. **(B)** The percentage of the major SNV classes identified in HCC samples. SNV classes are presented in y-axis. The percentage of each SNV type is in x-axis and the count is shown beside each bar. **(C)** The variant classification and their counts in HCC. **(D)** The top 10 mutated genes with their variant classifications. The color of classification is referred to **(C)**. The percentage of patients with each mutated gene is beside each bar. **(E)** The median of variants per sample is 38 among HCC samples with maximum of variants 569. **(F)** Detailed information of the top 15 mutated genes among 363 HCC patients. The upper panel illustrates the number of mutations of these top 15 genes of each sample shown in bar chart and the percentage of each mutated gene is shown on the right side.

In total, 374 TCGA HCC samples with clinical information were used for mutational landscape identification. There were more than 15,000 SNPs in HCC, taking into account the major variant type, including both transition and transversion. Among them, the substitution of thymine to cytosine, thymine to adenine, and cytosine to adenine showed relatively higher occurrence rates (Figures 1A,B). In addition, we found that the most dominant variant class, missense mutation, however, has a fluctuated proportion among different genes (Figures 1C,D). Overall, HCC patients presented a large fluctuation of variants with a median of 38 (Figure 1E). Besides, a highly heterogeneous distribution of variant types was observed in number of mutated genes among samples (Figure 1F). For example, 14% of HCC patients were with *CTNNB1* mutations and most were missense mutations, however, the composition of *TP53* mutations among the 14% mutated HCC patients were more diverse, in frame and frame shift mutations also contributed heavily in addition to missense mutation (Figure 1F).

These mutated genes exhibited diverse functions in HCC progression, by involving in multiple cancer related pathways, such as RTK-RAS, WNT, and NOTCH. However, the involved sample sizes for these biological processes were varied (Supplementary Figure S1). Even for the top mutated genes, only gene *MUC16* presented a significant difference of survival outcome between mutant and wild type groups (Supplementary Figure S2). The diverse single mutation types diluted the effect of the single mutated gene across different samples in HCC.

### Functional detection of co-mutated genes

**FIGURE 2:**
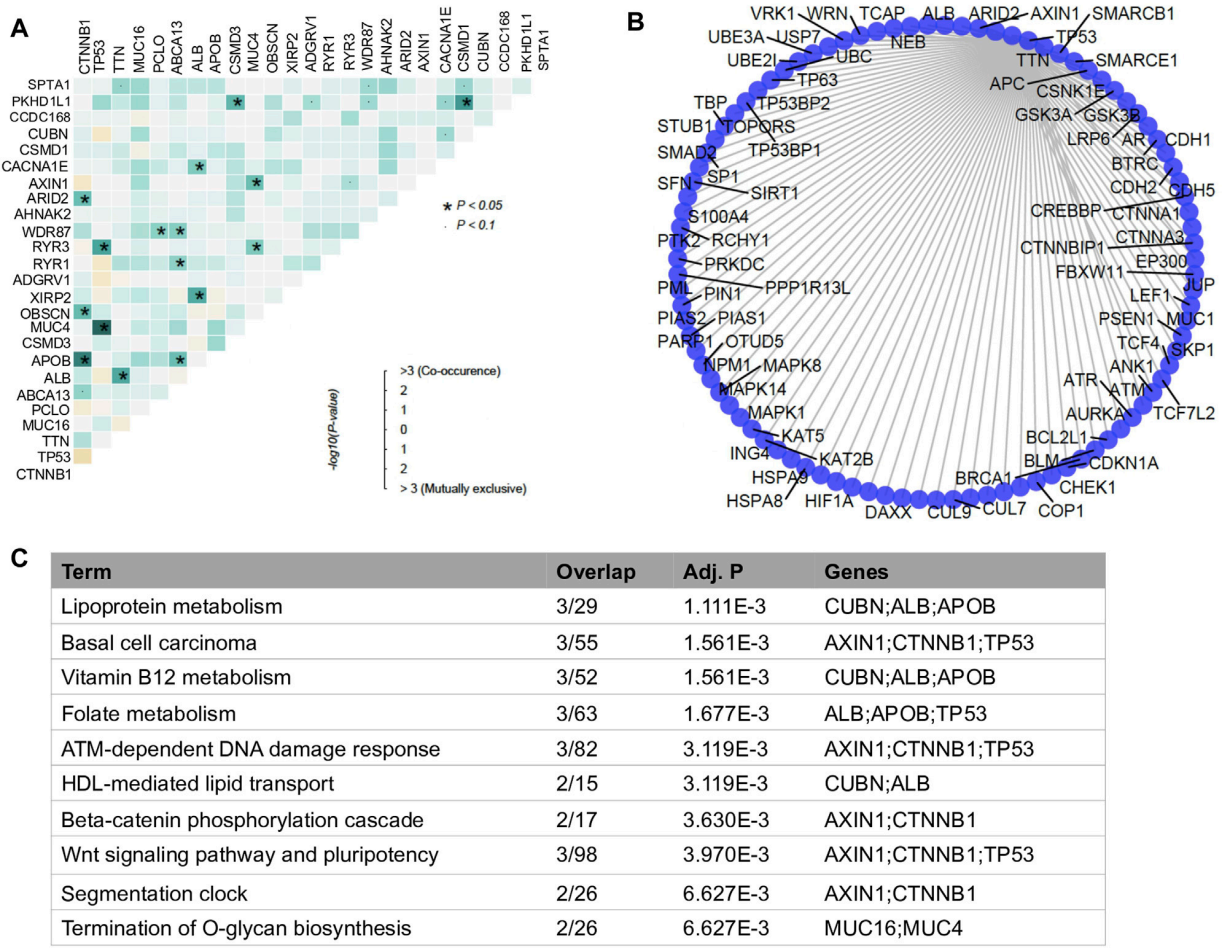
Analysis of co-mutated genes. **(A)** mutational co-occurrence in HCC. The color scale in grids represents the significance degree of the interaction of two genes. The darker the more significant. The dark blue square with asterisk represents the *p*-value of co-mutated gene pair is less than 0.05 and the light blue square with dot represents the *p*-value of co-mutated gene pair is less than 0.1. **(B)** Bar plot of enriched biological processes with −log10p >1 by databases of Reactome between variant and non-variant HCCs. **(C)** Functionally enriched biological processes of the co-mutated genes.

According to the co-occurrence analysis, we observed many cancer-related genes are co-mutated in HCC. Among them, the top 1 mutated gene *CTNNB1* significantly co-mutated with two other top mutated genes *APOB* and *OBSCN* (Figure 2A). However, similar to mutations of single genes, combinations of top mutated genes were also less predictive in survival outcome due to the heterogenicity of HCC. For instance, the co-occurrence of *APOB1* and *CTNNB1* mutations and the co-mutation of *TP53* and *MUC4* were observed in sample sets containing only 6 and 5 HCC patients, respectively (Supplementary Figures S3A,B). Therefore, in this study, we introduced the concept of four specialized mutational signatures to help understand the complexity of HCC (Supplementary Figure S4).

In addition, instead of focusing on the nature of mutated genes, we investigated the PPI network between co-mutated genes. We found that those genes closely interplayed with each other in their PPI network (Figure 2B). Two major functional clusters were enriched in the functional analysis, one was related to lipid metabolic pathways, including lipoprotein metabolism, vitamin B12 metabolism, folate metabolism and HDL-mediated lipid transport, the other category was relevant to signaling processes, such as ATM-dependent DNA damage response, beta-catenin phosphorylation cascade, and wnt signaling pathway and pluripotency (Figure 2C). This observation suggested that interaction among mutated genes may have led to the transcriptomic perturbation.

### Transcriptomic perturbation of coding genes

In this study, we utilized mutational signatures defined by COSMIC to help understand the complexity of HCC. By the COSMIC concept of mutational signature, four of them were detected specialized in HCC samples (Supplementary Figure S4). Among them, etiologies of SBS22 and SBS6 have been reported. SBS22 is found in cancer samples with known exposures to aristolochic acid (AA) and AA exposure has been reported to induce human liver cancers (Zhao L. et al., 2020). SBS6 is associated with defective DNA mismatch repair and is found in microsatellite unstable tumors (Alexandrov et al., 2020). Meanwhile, SBS12 and SBS40 are also closely related to cancers, although their etiologies are still not clearly identified, SBS12 contributes to a small proportion (<20%) of the mutations of liver cancer and SBS40 is correlated with patients’ ages of some cancers. Notably, liver cancer usually occurs among older people, its median diagnosis age is 67 years in males and 72 in females (Li et al., 2022).

**FIGURE 3:**
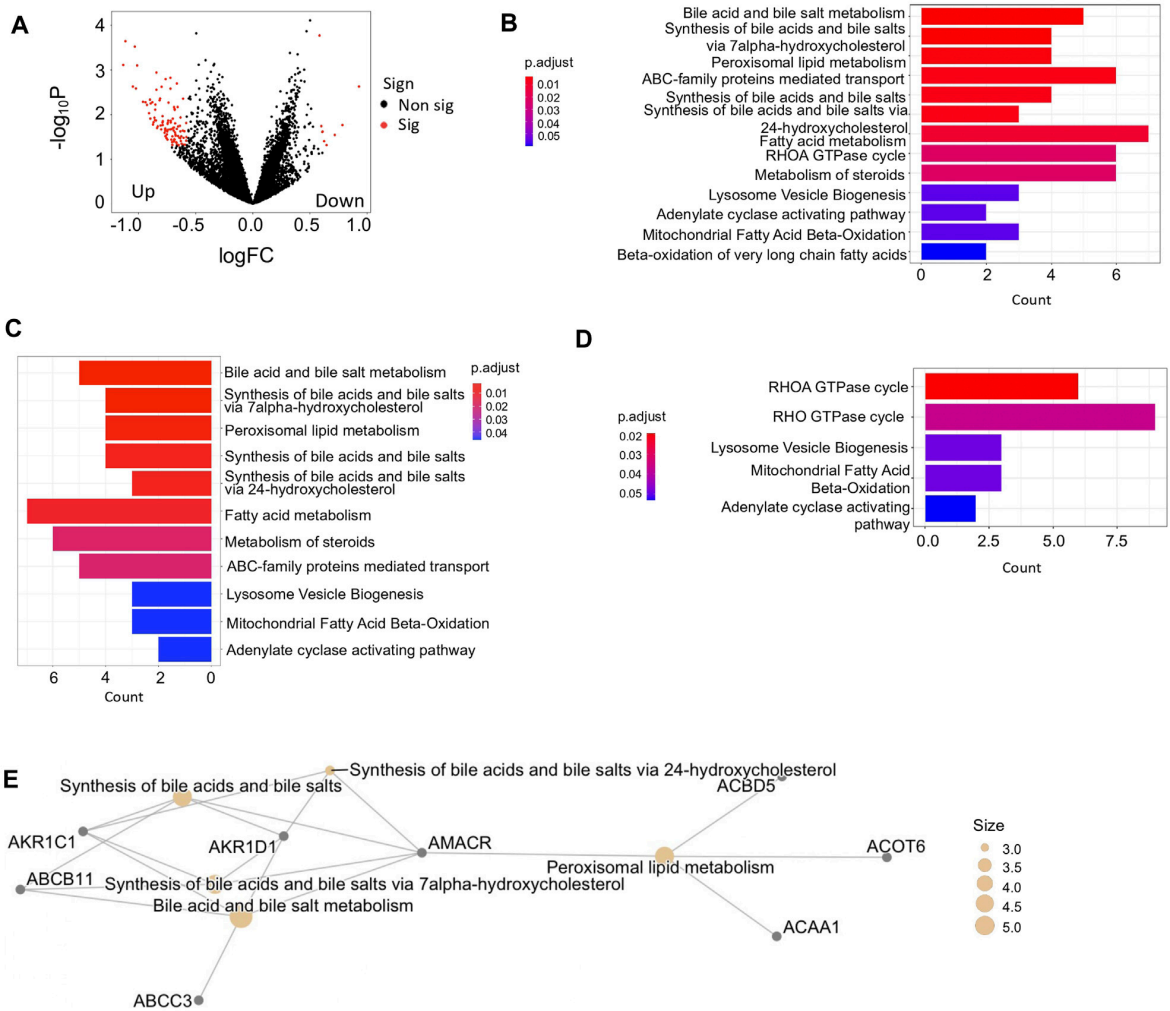
Analysis of coding gene expression between variant and non-variant HCCs. **(A)** Volcano plot of DEGs. logFC <0 represents upregulated genes in variant HCCs compared with non-variant HCCs. Red color represents the dots pass the filtering criteria i.e., |logFC| <0.5 and -log10p >1. **(B)** Bar plot enriched biological processes with -log10p >1 by databases of Reactome between variant and non-variant HCCs. **(C,D)** Bar plot of enriched pathways of upregulated genes **(C)** and downregulated genes **(D)** in variant HCCs compared with non-variant HCCs. **(E)** The pathway network of top 5 enriched pathways enriched by upregulated genes in variant HCCs compared with non-variant HCCs.

Patients exhibiting at least one mutational signature were categorized into variant group, whereas the rest patients were grouped as the non-variant HCC sample set. Indeed, we found 112 significantly DEGs between two groups, including 103 upregulated genes and 9 downregulated genes in the variant group (Figure 3A). These DEGs were found involving in versatile functional processes during tumor development (Figure 3B). Based on cell marker information collected from database CellMarker (Zhang et al., 2019), we observed marker genes of diverse infiltrated immune cells also significantly differentially expressed between variant and non-variant HCCs during tumor development Among these immune marker genes, exhausted CD4^+^ and CD8^+^ T cells accounted for critical proportions (Supplementary Materials). T cell exhaustion results in impaired effector function whereby cytotoxic CD8^+^T cells fail to control tumor progression, especially in the late stage (Weiqin et al., 2021).

In addition, the upregulated DEGs mostly concentrated in lipid metabolism related functions (Figure 3C) and the downregulated DEGs participated in the processes toward the tumor microenvironment (Figure 3D). The upregulated DEGs enriched pathways were highly connected by their shared genes, which are focused on bile acid and bile salt metabolism (Figure 3E), however, not like upregulation, the downregulated DEGs enriched pathways were not concentrated, due to the insufficient DEG numbers.

Moreover, several genes were capable of serving as indicators of prognostic risk and some of them were also DEGs between variant and non-variant HCCs. For example, the overexpression of genes *CPSF6*, *LOC151174*, *CYP26B1*, and *GPR83* were more likely associated with poor survival outcomes in variant HCC patients, among them, *CPSF6* and *GPR83* were also DEGs between variant and non-variant HCCs (Supplementary Figure S5).

### Transcriptomic perturbation of miRNAs

**FIGURE 4:**
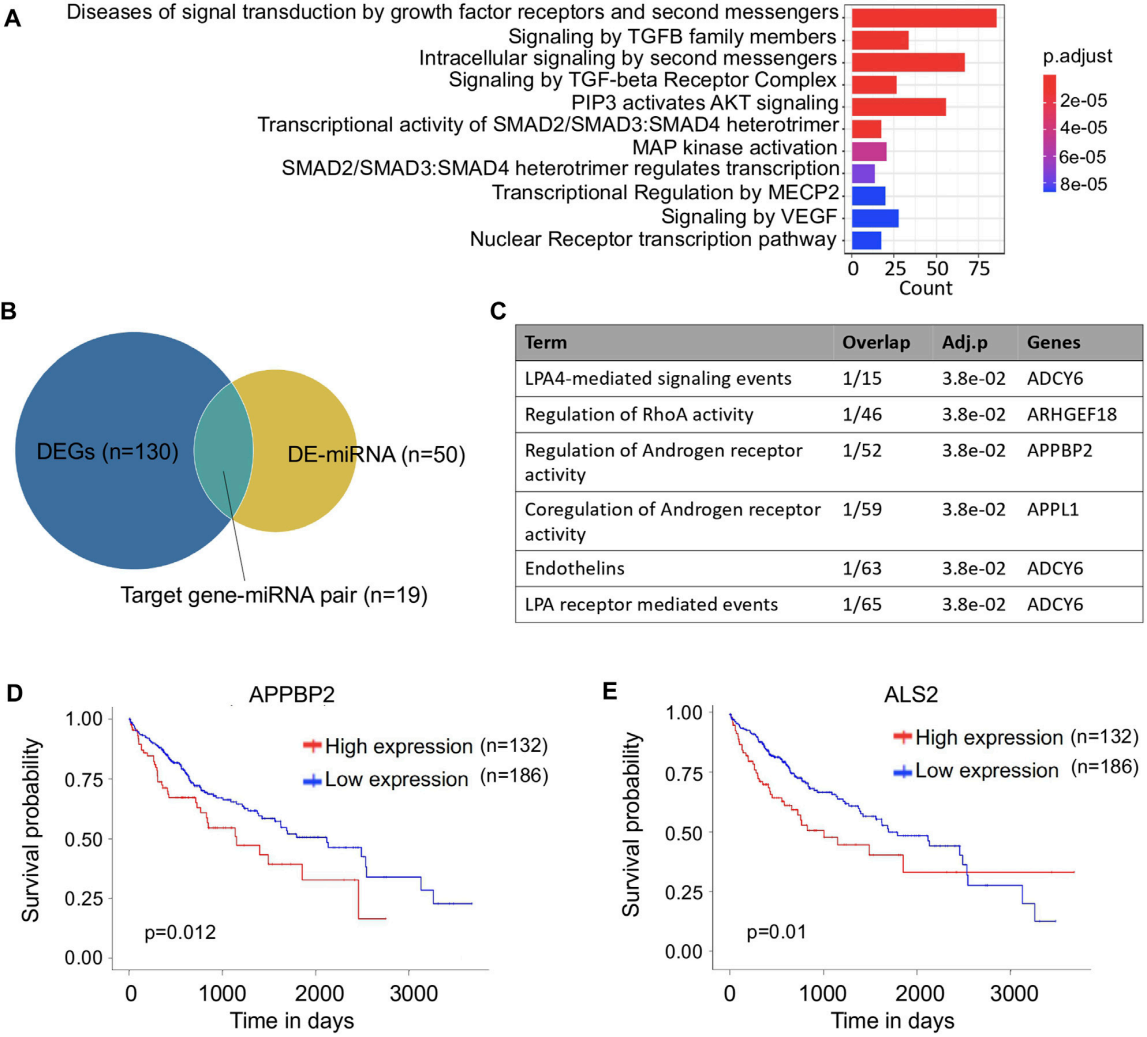
Analysis of miRNA between variant and non-variant HCCs. **(A)** Bar plot of enriched pathways of differentially expressed miRNA target genes. **(B)** Venn plot of differentially expressed mRNAs and miRNAs. **(C)** Functionally enriched biological processes of the miRNA target genes. **(D,E)** Kaplan–Meier survival curves of *APPBP2* **(D)** and *ALS2* **(E)** for overall survival of HCC patients.

In addition to the coding genes, non-coding RNAs also contributed to the transcriptomic changes. As a crucial epigenetic regulator, miRNA plays a key role to regulate the expression of target genes during tumor development (Yong and Carlo, 2016). In the variant HCC group, the target genes of differentially expressed miRNAs (DEmiRNAs) were enriched in cancer related signaling pathways. Among them, the essential intracellular components Smad family members that involved in TGF-β relevant signaling processes were of special interest (Figure 4A). From previous studies, disorganization of TGF-β signaling is associated with a growing incidence of HCC, however, overexpression of signaling transducer Smad3 can reduce the susceptibility of HCC (Yang et al., 2006). Our study provided evidence that the miRNAs in variant HCCs probably participated in this regulation process.

Among the miRNA-target gene pairs, 19 differentially expressed pairs were identified in the variant HCC group (Figure 4B **)**, and one of the miRNA-targets, *APPBP2* was found involving in androgen regulatory processes (Figure 4C). In HCC, the incidence of males is three to four times higher than in females (Chacko and Samanta, 2016), thus *APPBP2* probably can be used as potential therapeutic target for HCC treatment. Moreover, significantly different survival outcomes were associated with the differential expression of two target genes, *APPBP2* and *ALS2*, suggesting their potential to serve as prognostic indicators in HCC treatment. Interestingly, the sample with the longest follow-up time was with a high expression level of *ALS2*, while more samples were with low expression levels, which leads to a survival curve cross between its high and low levels (Figures 4D,E). In addition, by searching The Human Protein Atlas database (Mathias et al., 2005), immunohistochemical staining for *APPBP2* was positive in HCC based on the immunohistochemistry (IHC) results (Supplementary Figure S6), as well it particularly expressed in endothelial and hepatic stellate cells referring to the cell type specific analysis (Supplementary Figure S7 and Supplementary Material).

### Deregulation of ceRNA regulatory network through lncRNA in variant HCC

As another critical regulator of non-coding RNA, lncRNAs play suppressive and oncogenic roles during HCC tumorigenesis (Zhao H. et al., 2020). They indirectly regulate the expressions of coding genes through competitively shared miRNAs. In the variant HCC group, 90 differentially expressed lncRNAs (DElncRNAs) were identified when comparing the variant HCC with non-variant HCC group. However, no statistically significant function was enriched according to Supplementary Table S1.

**FIGURE 5:**
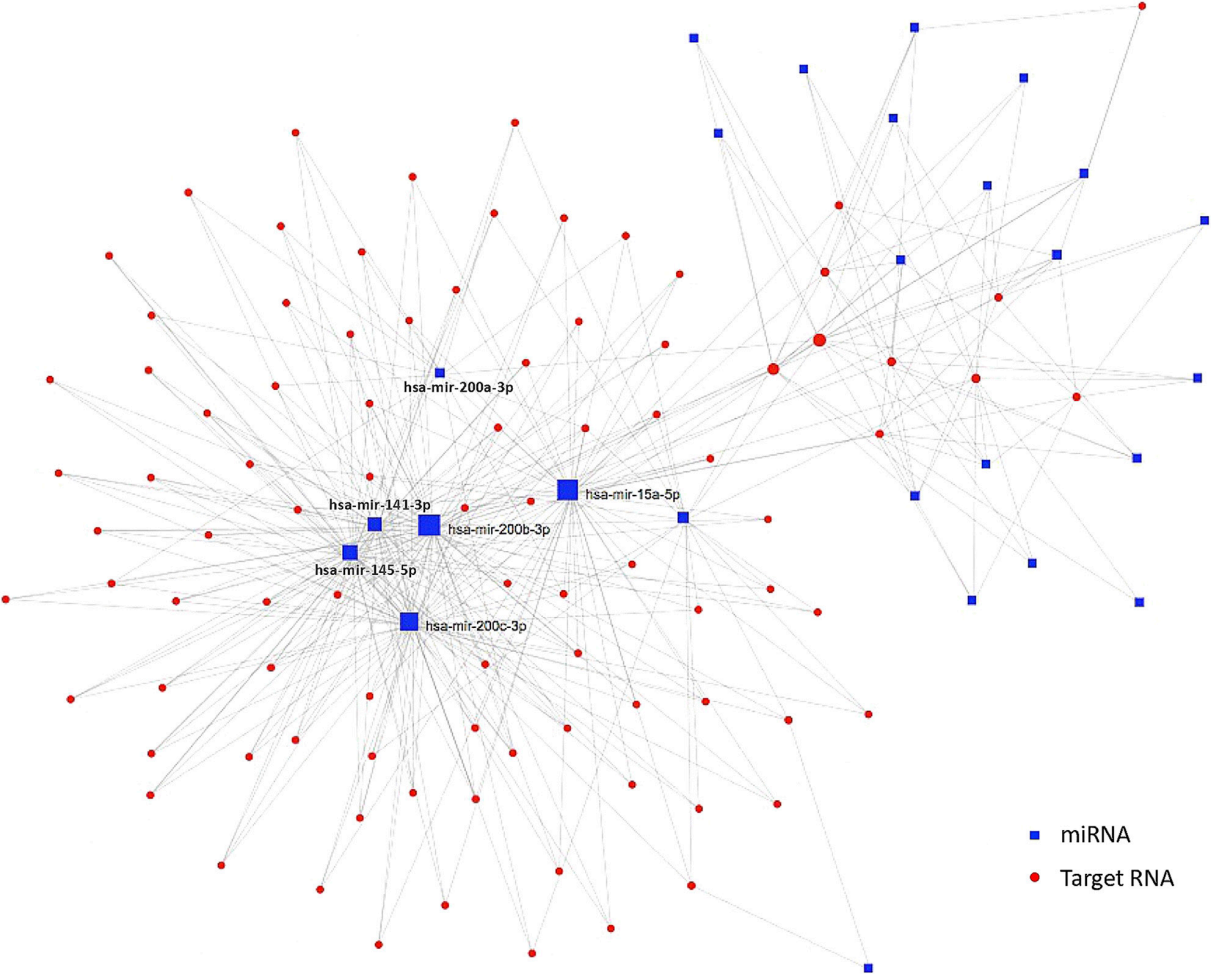
ceRNA regulatory network in variant HCCs. Blue squares are significantly differentially expressed miRNAs and the red dots are their targets. Those miRNAs condensed connected with target genes are labeled beside corresponding blue squares.

According to a previous HCC study which utilized TCGA RNA data, a lncRNA-miRNA-mRNA network has been identified in tumor samples in the comparison with non-tumor samples (Wang et al., 2021). However, the lack of significant DEmiRNA-lncRNA connection in variant HCCs in this study weakened the ceRNA regulatory network, which only composes of DEmiRNAs and their corresponding target coding genes (Figure 5). The indirect regulations of miRNAs target genes through lncRNAs were eliminated from the ceRNA regulatory network of variant HCCs, indicating that the targeted inhibition of miRNAs probably is an attempt therapy for variant HCCs.

## Discussion

HCC has become the second leading death malignancy in the world, and moreover, its incidence stably increases every year (Li et al., 2019). Currently, although surgical resection and liver transplantation have been utilized for HCC early stage, the five-year overall survival rate is far from satisfaction due to its complicated and heterogenous molecular etiologies (Jordi and Josep, 2009). Consequently, it is urgent to identify potential therapeutic and prognostic indicative genes associated with complex pathogenesis for HCC treatment. Numerous studies have identified mutational effects on HCC through many critical functional progresses (Lee, 2015), as well as aberrant expression of mRNA (Delia et al., 2018), miRNA (Xin et al., 2018), and lncRNA (Chacko and Samanta, 2016). Nonetheless, few studies have linked them together to explore their crosstalk relationships and projections between multilayers of molecular landscapes in HCC, thus a systematic study for them is urgently required.

In this study, mutational signatures of HCC were discovered, furthermore, more specifically, some potential cancer markers with significantly aberrant expressions were found co-mutated in HCC. For example, *MUC4* has been recognized as a prognostic factor of Cholangiocarcinoma (CC) by several studies (Hiroaki et al., 2004; Li et al., 2016). *TTN*, a potential skin cutaneous melanoma related marker (Ying et al., 2020), was co-mutated with *MUC4* in HCC based on our analysis. Another example of co-mutation identified in this study was *APOB* and *CTNNB1,* which are two potential markers of HCC (Lee et al., 2018; Davod et al., 2020). These observations indicated mutational changes co-occurred in multiple critical genes probably induce expression alterations of themselves, and the genes directly and indirectly regulated by them.

Expression profiles of mRNA, miRNA, and lncRNA for HCCs with mutational signatures were identified in the comparison with HCCs without mutational signatures using TCGA data. The DEGs for different types of RNA were associated with specific biological functions during HCC development. For example, upregulated coding RNAs in the variant HCC group were predominantly enriched in lipid metabolism related functions, whereas the downregulated coding RNAs were enriched in axonogenesis for tumor microenvironment generation. Additionally, the DEmiRNAs were inclined to enrich in cancer related signaling pathways.

A portion of these DEGs also possessed the potential to serve as prognostic indicators to predict the survival outcome of HCCs with mutational signatures, such as the high levels of expression of *CPSF6*, *LOC151174*, *CYP26B1,* and *GPR83* were associated with poor patient outcomes of HCC patients. The overexpression of *CPSF6* is clinically identified in human breast cancer, moreover, its expression correlates with poor outcomes of patient (Najat et al., 2017). Similarly, increased expression of *CYP26B1* is observed in 25.2% of tumors and is significantly diseased expressed in normal colonic epithelium (*p* < 0.001), furthermore, its enhanced expression is also significantly associated with poor prognosis (Gordon et al., 2014). In cancers, many processes also involve in immune response. For example, the high expression of GPR83 regulated by CD4^+^CD25^+^ regulatory T cells (Tregs) participants in the induction of CD4^+^Foxp3^+^ Tregs in the course of an ongoing immune response (Hansen et al., 2010).

Hepatitis viruses are critical risk factors of HCC and some of them could integrate their genes into the human genome. However, in this study, we didn’t observe enough evidence to support the integrated genes altered between variant and non-variant HCCs (Supplementary Materials). Although 100 previously reported integrated genes (Hayer et al., 2013) were involved in our DEGs, but none of them had significant integration *p* value, which indicates its integration probably is an occasional event and its effect on differential expression between variant and non-variant HCCs requires further investigation.

We assembled RNA regulatory network integrating miRNAs and their target RNAs to pinpoint the RNAs with regulatory relationships with others. Rapidly emerging evidence proved that ceRNAs play critical roles in tumorigenesis. The expression of RNA transcripts is regulated by other ceRNAs through the common miRNA shared by them (Qi et al., 2015). For instance, lncRNA LINC00668 competingly regulates gene VEGF-A through their shared miRNA miR-297 to strengthen cell proliferation ability in the oral squamous cell (Zhang, 2017). As well as increased expression of H19 lncRNA enhances the expression of VASH2 through the common miRNA miR-29a (Zheng et al., 2018). In this study, the mutational signatures led to significant miRNA-mRNA alterations in variant HCCs, and few significant miRNA-lncRNA changes were identified. Thus, the inhibitive regulation *via* lncRNAs were lost. The recovery of inhibition by target miRNAs provided another possible therapeutic way for HCCs with mutational signatures. Furthermore, this potential miRNA targeting treatment could reduce the complexity due to the extremely diverse mutation profiles and signatures. Therefore, our multiomics analysis not only identified the altered relationships between omics, but also provided a projection from mutational signatures to transcriptomic changes, which affords potentially easier therapeutic approaches.

## Data Availability

The original contributions presented in the study are included in the article/Supplementary Material, further inquiries can be directed to the corresponding authors.
