# Supplementary material for: Mutational signatures representative transcriptomic perturbations in hepatocellular carcinoma
Source: Front Genet. 2022 Aug 23;13:970907. doi: 10.3389/fgene.2022.970907 (PMC9445436; doi:10.3389/fgene.2022.970907)
Supplement: Supplementary file 1 [file DataSheet4.PDF]

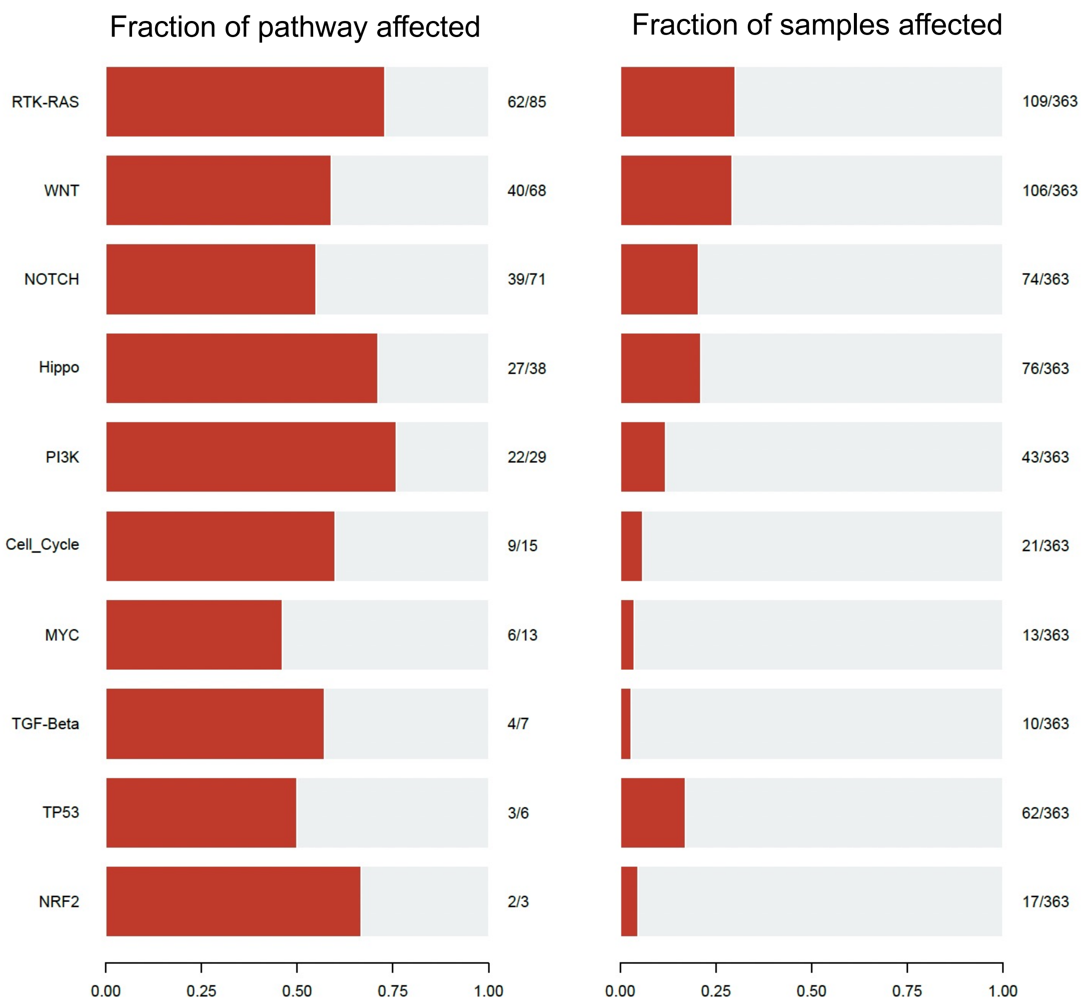

Figure S1. The correspondence between affected pathways (left) and HCC samples (right).

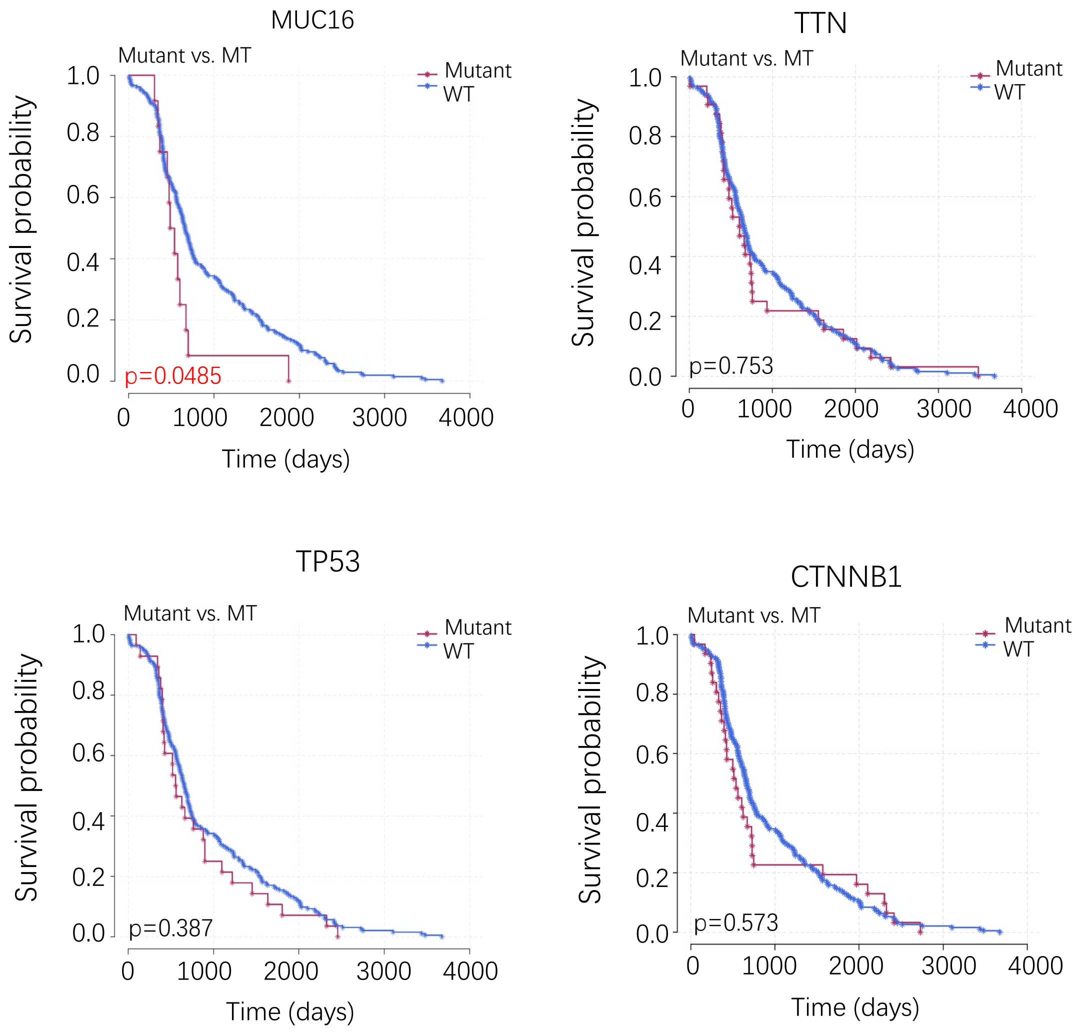

Figure S2. Kaplan–Meier survival curves for overall survival of HCC patients of mutated single genes.

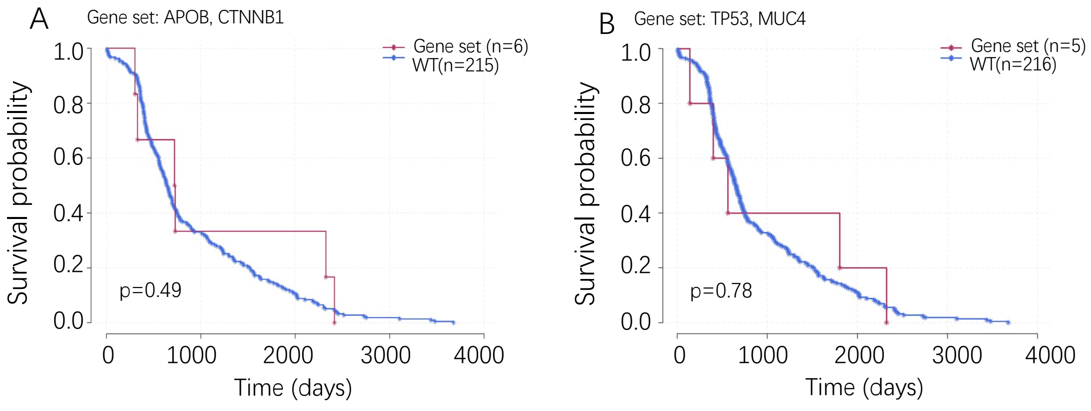

Figure S3. Kaplan–Meier survival curves for overall survival of HCC patients of co-mutated genes.

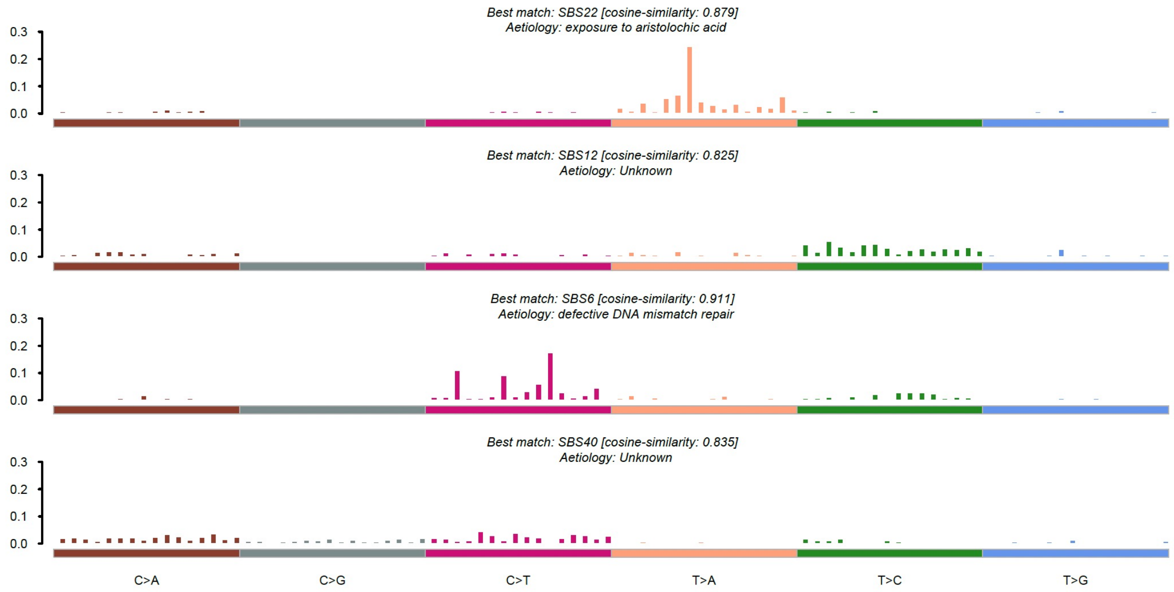

Figure S4. Top 4 COSMIC mutational signatures of HCC samples.

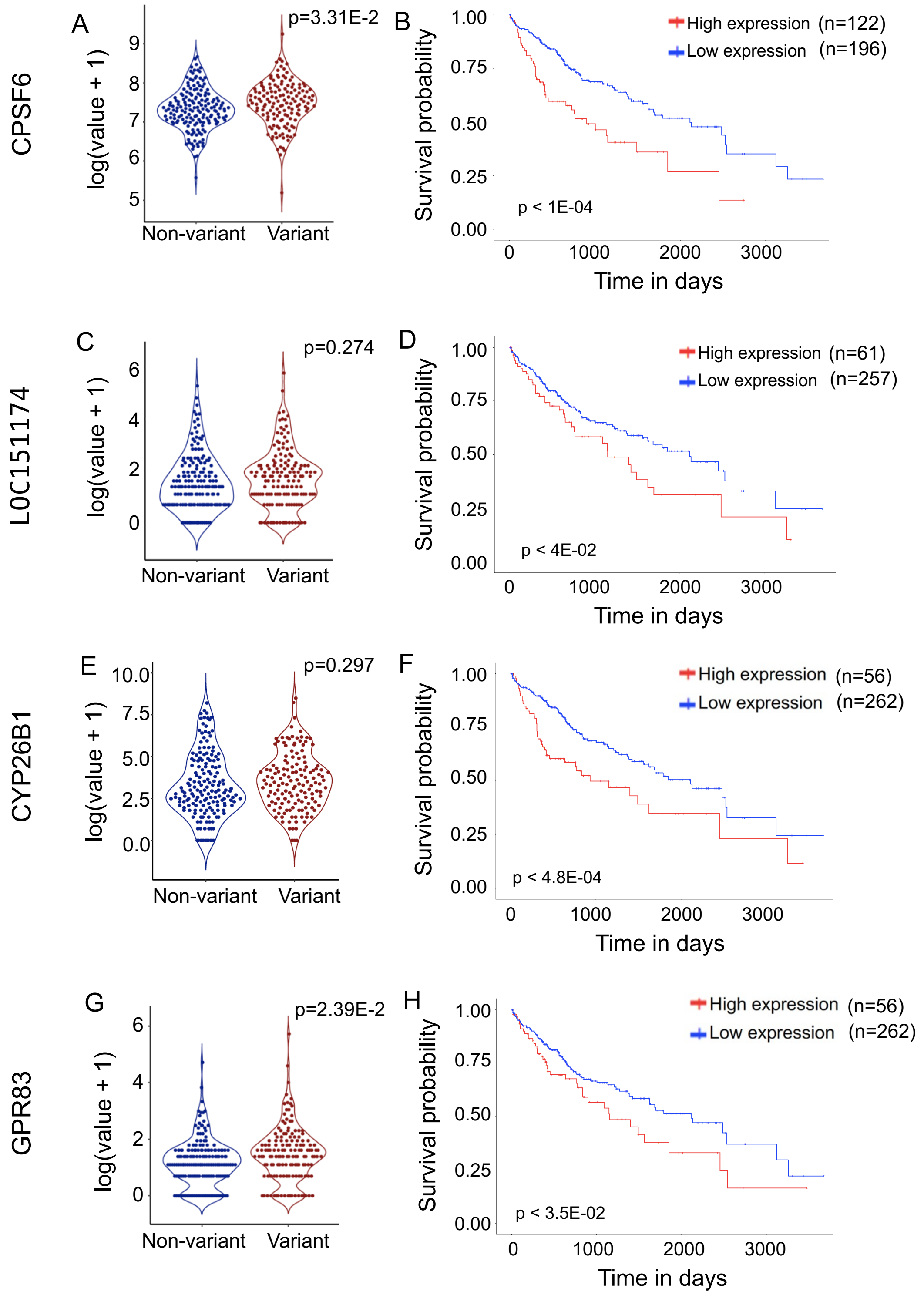

Figure S5. Differentially expressed genes and their Kaplan–Meier survival curves for overall survival of HCC patients.

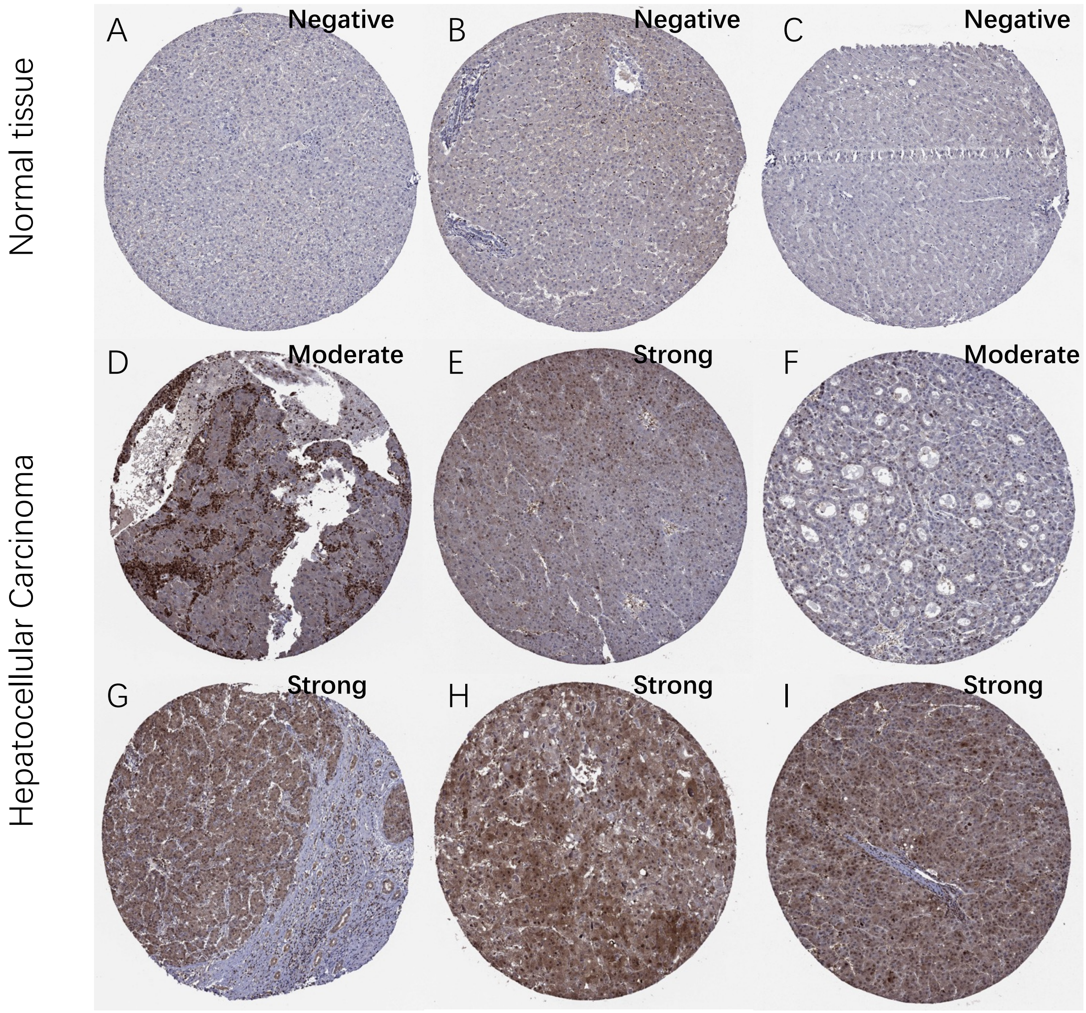

Figure S6. Representative images of normal and HCC tissue samples labelled with IHC for *APPBP2*. (**A-C**) Antibody staining images of human normal liver tissue samples of female, age 32 (**A**), female, age 50 (**B**) and male, age 67 (**C**). (**D-I**) Antibody staining images of human HCC tissue samples of male, age 75 (**D**), male, age 67 (**E**), male, age 70 (**F**), male, age 65 (**G**), female, age 73 (**H**) and female, age 82 (**I**). Antibody is HPA078370. Staining intensity for each sample is labeled in the top-right corner of each IHC image.

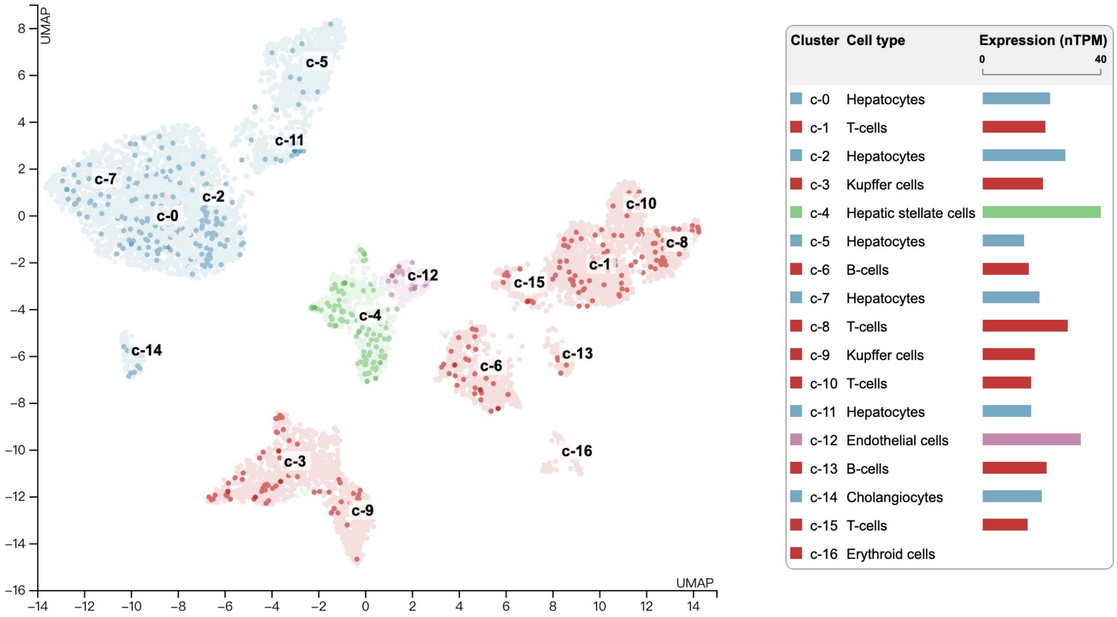

Figure S7. Scatter plot of cell types in liver tissue. Colored according to cell type group.

Table S1. Differentially expressed lncRNAs related biological processes.

Index	Name	P-value	Adjusted p-value	Odds Ratio	Combined score
1	Interleukin-2 Receptor Alpha Subunit	0.03544	1.000	31.95	106.70
2	Proteinuria	0.07801	1.000	13.15	33.54
3	Epilepsy	0.04282	1.000	6.35	20.01
4	Energy Metabolism	0.1147	1.000	8.59	18.61
5	Chemical And Drug Induced Liver Injury	0.2129	1.000	4.29	6.64
6	Psychotic Disorders	0.2129	1.000	4.29	6.64
7	Warfarin	0.2235	1.000	4.06	6.08
8	Interleukin-10	0.2270	1.000	3.98	5.91
9	Alpha-Carotene	0.2374	1.000	3.78	5.44
10	Tuberculosis	0.2612	1.000	3.38	4.54
